# Changes in Smoking Patterns in Turkey Before and After the Ban on Loose Cigarettes: An Ecological Perspective From Global Tobacco Surveillance Data

**DOI:** 10.7759/cureus.69657

**Published:** 2024-09-18

**Authors:** Nancy Satpathy, Pratap K Jena, Monalisha Sahoo, Sonam J Agarwal, Krutideepa Raut, Manasmruti Sahu, Aishwarya Garnaik, Soumini Samal, Subhalaxmi Bal, Arpita Patel

**Affiliations:** 1 Public Health, Indian Council of Medical Research (ICMR), New Delhi, IND; 2 Community Medicine, Institute of Medical Sciences and SUM Hospital, Siksha 'O' Anusandhan Deemed to be University, Bhubaneswar, IND; 3 Public Health, Kalinga Institute of Industrial Technology (KIIT) School of Public Health, Bhubaneswar, IND; 4 Healthcare Management, Swiss School of Business and Management (SSBM Geneva), Geneva, CHE; 5 Public Health Dentistry, Faculty of Dental Science, King George's Medical University (KGMU), Lucknow, IND; 6 Public Health, Kalinga Institute of Industrial Technology (KIIT) School of Public health, Bhubaneswar, IND; 7 Public Health, National Law University Odisha, Cuttack, IND; 8 Dentistry, Hi-Tech Dental College & Hospital, Bhubaneswar, IND

**Keywords:** global adult tobacco survey (gats), loose cigarettes, smoking patterns, tobacco control policy, turkey

## Abstract

Introduction: Loose or individual cigarettes sold without packaging pose a unique challenge for tobacco control, which is the leading cause of premature and preventable mortality worldwide. This study aims to assess the changes in smoking patterns following the ban on loose cigarettes in 2008 in Turkey.

Method: Gender-stratified trend analysis of smoking patterns before and after the ban on loose cigarettes was done using the Global Tobacco Surveillance data (adults: Global Adult Tobacco Survey (GATS) 2008, 2012, and 2016; youth: Global Youth Tobacco Survey (GYTS) 2003, 2005, 2009, 2012, and 2017) from Turkey. The trends in smoking patterns were triangulated with the introduction of the loose cigarette ban and other laws in Turkey.

Result: From GATS 1 in 2008 to GATS 2 in 2012, there were initial declines in adult smoking patterns, including current smoking and daily smoking, with increased quit intentions. However, these trends were reversed by the time of the GATS 3 in 2016, when both current smoking and daily smoking measures were higher, especially among females. Monthly cigarette expenses consistently increased across all survey years. Among youths, the overall prevalence increased sharply from 6.9% in 2003 to 23% in 2005, then decreased to 8.4% in 2009, again increased to 10.4% in 2012, and finally returned to 7.7% in 2017. Triangulation of these trends against the implementation of the loose cigarette ban in 2008 produced no clear association between the ban and smoking patterns.

Conclusion: The overall marginal decline in smoking behaviors over two decades in Turkey needs to be fast-tracked, specifically the rise of smoking among the female population, which needs to be addressed through evidence-based tobacco control interventions. The loose cigarette ban was implemented as part of a comprehensive tobacco control law, and the GATS or GYTS data is not sufficient to assess the effect of this provision. The tobacco control effort in Turkey should collect evidence for changes in smoking patterns following the implementation of tobacco interventions, which is also a mandate of the Global Tobacco Surveillance System (GTSS).

## Introduction

Tobacco use remains a significant global public health concern, causing over eight million deaths annually [1]. One of the contributing factors to this challenge is the availability of loose cigarettes, which are individual cigarettes sold without packaging [2]. These products exacerbate the tobacco epidemic by targeting vulnerable populations facing affordability and accessibility challenges [3]. Compared with packaged cigarettes, the lower cost of loose cigarettes makes them particularly appealing to individuals with limited financial resources [4]. Furthermore, the lack of standardized labeling and regulatory oversight associated with loose cigarettes fosters illicit trade, posing a formidable challenge to broader tobacco control efforts [5].

Turkey has a deep-rooted history of tobacco use, particularly among men, dating back to Ottoman times [6]. The expression "Smoking like a Turk" once echoed in various European languages, highlighting its cultural significance [6]. In addition to being an addictive consumable, tobacco has also been a substantial revenue source, attracting foreign investors because of its lucrative market potential [7]. Traditionally, gatherings in coffee houses involved socializing, gaming, and smoking, initially with hand-rolled cigarettes and water pipes (narghile) [8]. The Turkish folk poet Neşet Ertaş depicted smoking as a "stress relief method" and a "simple pleasure for the poor," reflecting societal acceptance [9].

Turkey has taken extensive measures to combat tobacco use and its associated health risks through a comprehensive approach. This approach encompasses various strategies, including legislation, public awareness campaigns, smoking cessation support, taxation policies, enforcement, monitoring, and stakeholder engagement [10]. Actions such as enacting bans on tobacco advertising, promotion, and sponsorship; implementing plain packaging laws; enforcing smoke-free policies in public spaces; running media campaigns; establishing smoking cessation clinics and helplines; and increasing taxes on tobacco products have been instrumental [10,11]. Turkey has effectively aligned with the MPOWER (Monitor tobacco use, Protect people from tobacco smoke, Offer help to quit, Warn about dangers of tobacco, Enforce bans on tobacco advertising, Promotion and sponsorship, and Raise tobacco tax) strategies outlined by the World Health Organization (WHO), which involve monitoring tobacco use, protecting individuals from smoking, supporting cessation efforts, warning about tobacco dangers, enforcing bans, and raising taxes on tobacco products. Key stakeholders, such as the Turkey Ministry of Health and non-governmental organizations, have played pivotal roles in these comprehensive efforts [12].

Despite these initiatives, Turkey faces challenges such as high smoking rates, gender disparities, the popularity of non-cigarette tobacco products, smuggling, narghile use, and an uneven distribution of cessation centers [13]. Of particular concern are loose cigarettes, which significantly contribute to smoking prevalence and related health issues [14]. Even with extensive tobacco control measures in place, Turkey remains the third highest consumer of tobacco among Organization for Economic Co-operation and Development (OECD) countries [15]. Turkey implemented nationwide bans on loose cigarette sales in 2008; however, the specific association of these bans within Turkey's unique context remains inadequately explored, necessitating further investigation.

Understanding smoking patterns requires a multifaceted analysis that incorporates various key variables. Current smoking directly reflects the prevalence of smoking in a population at a given time, indicating the scale of tobacco use. Daily smoking provides insight into the frequency of smoking among individuals, revealing the regularity of smoking habits. Quitting attempts and future quitting shed light on cessation efforts and intentions, offering perspectives on individuals' readiness to quit smoking. These variables collectively contribute to shaping smoking patterns by influencing the number of active smokers and the dynamics of smoking cessation within a population [16]. Additionally, the average monthly expenditure on cigarettes adds an economic dimension, highlighting the financial aspect of smoking and its potential influence on consumption behaviors. The relationships between the number of loose cigarettes and these critical indicators are particularly significant. Loose cigarettes, which are available in relatively small quantities, play a pivotal role in facilitating daily smoking habits and may attract price-sensitive populations, affecting both smoking prevalence and expenditure [17]. Furthermore, the accessibility of loose cigarettes can influence individuals' readiness to quit, potentially hindering quit attempts and future intentions. Recognizing and exploring this linkage is crucial for understanding the changes in smoking patterns in Turkey before and after the ban on loose cigarettes.

This study aimed to assess the changes in select smoking patterns among males and females following the ban on loose cigarettes in Turkey using data from the Global Adult Tobacco Survey (GATS) and Global Youth Tobacco Survey (GYTS).

## Materials and methods

This is an ecological study design relating loose cigarette ban on smoking behavior in Turkey, using data from the national surveys of GATS 2008, 2012, and 2016, and GYTS 2003, 2005, 2009, 2012, and 2017. In this study, information from three GATS and five GYTS were used to identify gender-stratified changes in smoking patterns following the loose cigarette ban in Turkey.

Both the GATS and GYTS are nationally representative cross-sectional surveys and are part of the Global Tobacco Surveillance System (GTSS) [18]. The GATS enrolls individuals 15 years and above in a household survey and conducts personal interviews to collect data [19]. The GYTS is a school-based survey that enrolls children aged 13-15 years and collects data through self-administration [20]. These surveys use standardized protocols and data collection tools and multistage and geographically clustered sampling techniques for collecting data used for monitoring tobacco use and the associations of various policy interventions. The sample weights are computed in each of the survey years to derive representative estimates.

The variables from the GATS datasets considered in this study were current smoking, quit attempt, quit intention, daily smoking, and monthly average expenditures on cigarettes. From the GYTS datasets, only the youth current smoking status was considered. In this study, country-level information on male and female smoking patterns from three GATS and five GYTS fact sheets was used to identify gender-stratified changes in smoking patterns in relation to the ban on loose cigarettes in Turkey. The clustered bar diagram with a trend line was generated with the help of a specialized AI tool, ChatGPT-4, configured as "Diagrams & Data: Research, Analyze, Visualize," developed by OpenAI (San Francisco, CA). The Python code generated and steps followed during such analysis are given in the Appendices (Supplementary 1).

Furthermore, the trend in smoking patterns was triangulated with policy interventions in Turkey. These interventions were expected to be detected as effective by comparing the changes in smoking patterns before and after the execution of key tobacco control policies, which include a prohibition on the sale of loose cigarettes. This study is based on publicly available, anonymous, and country-level secondary data from the GATS and GYTS fact sheets without any direct interaction with humans, which ensures the anonymity of the participants; hence, no separate ethical approval was taken.

## Results

Smoking pattern and its trend in Turkey

Among adults, the current smoking rate was 30.1% in 2008, 26.9% in 2012, and 31.6% in 2016, indicating an overall increase in smoking prevalence from 2008 to 2016 in GATS 3, the third survey. This represents an increase of 4.2 percentage points from GATS 2 and 1.5 percentage points from GATS 1, as indicated in Table 1.

**Table 1:** Overall changes in adult smoking patterns and monthly expenditures on cigarettes in Turkey GATS: Global Adult Tobacco Survey Source: Global Tobacco Surveillance System Data (GTSSData) [21]

Indicator	GATS 1 (2008)	GATS2 (2012)	GATS 3 (2016)
Current smoking	30.1%	26.9%	31.6%
Daily smoking	27.4%	23.8%	29.5%
Quit attempt	46.9%	46%	24.6%
Future quit	53%	55.2%	32.8%
Average monthly expenses on cigarettes (Turkish currency)	86.5	146.1	264.4

**Figure 1:**
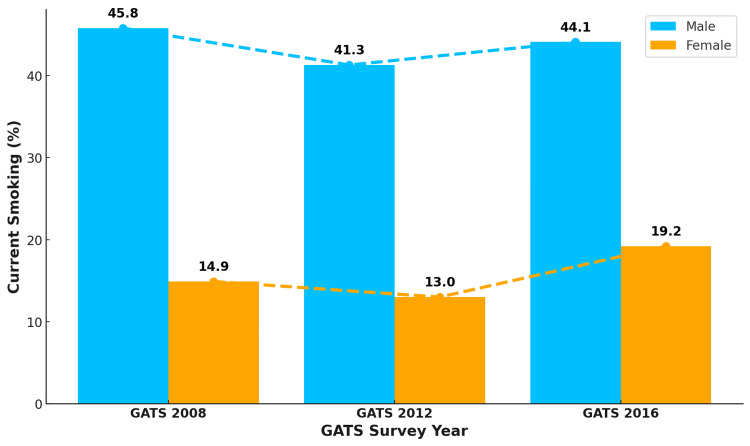
Trend in current cigarette smoking patterns among males and females in Turkey The numbers in the figure are in percentage (%). GATS: Global Adult Tobacco Survey

In all three rounds of GATS, males outnumbered females in current smoking. The current smoking rates for males were 45.8%, 41.3%, and 44.1% in the first, second, and third rounds of the GATS, respectively, indicating a decline from 2008 to 2012, a rise from 2012 to 2016, and an overall marginal decline in the male smoking pattern from 2008 to 2016. The female current smoking rates were 14.9%, 13%, and 19.2% in the first, second, and third rounds of GATS, respectively, suggesting a similar trend as that reported among males, but an overall increase in prevalence of 4.3 percentage points from 2008 to 2016 was observed, as shown in Figure 1.

**Figure 2:**
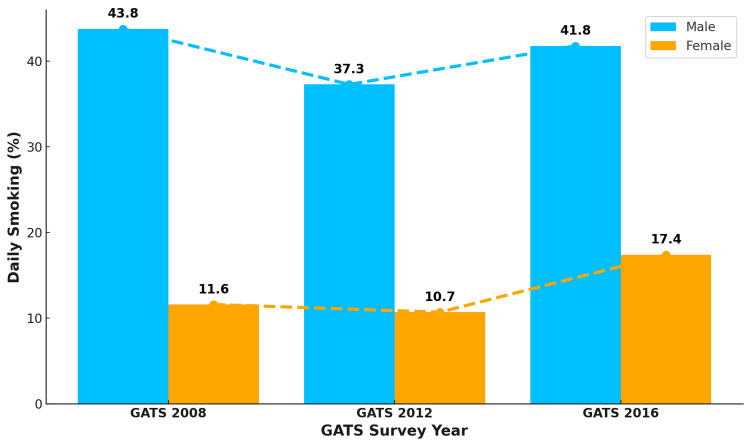
Trend in daily cigarette smoking patterns among males and females in Turkey The numbers in the figure are in percentage (%). GATS: Global Adult Tobacco Survey

Males outnumbered females in daily smoking in all three rounds of GATS. Daily smoking among males and females followed a similar trend to that of current smoking, with an overall 2.0 percentage point decline among males and a 5.8 percentage point increase among females from 2008 to 2016, as shown in Figure 2.

**Figure 3:**
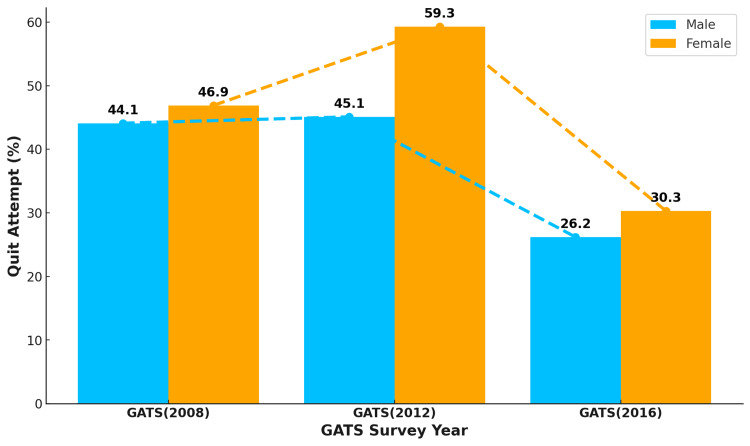
Trend in quit attempts among current male and female cigarette smokers in Turkey The numbers in the figure are in percentage (%). GATS: Global Adult Tobacco Survey

The proportion of male current smokers making a quit attempt increased from 44.1% by 1.0 percentage points from 2008 to 2012 and decreased to 26.2% in 2016. The trend in quit attempts among female current smokers followed a similar pattern but with marked differences. The number of quit attempts among females remained greater than that among their male counterparts in all three rounds of GATS, as shown in Figure 3.

**Figure 4:**
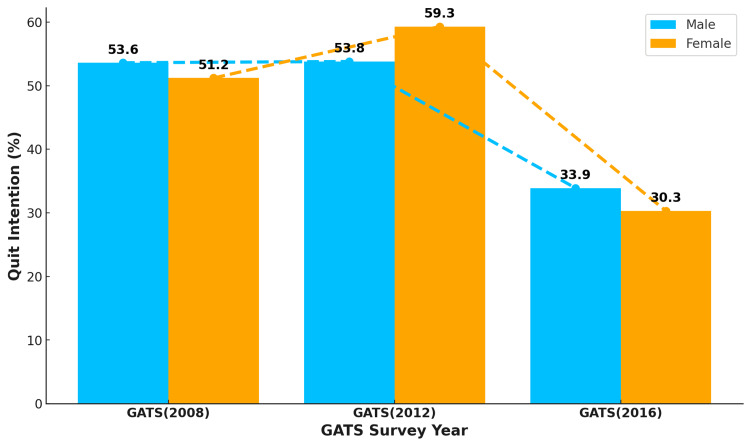
Trend in quit intentions among current male and female cigarette smokers in Turkey The numbers in the figure are in percentage (%). GATS: Global Adult Tobacco Survey

Smoking quit intentions among males were greater than those among females in the first and third rounds of the GATS (Figure 4), whereas the reverse pattern was observed in the second round. During the first and second rounds of the GATS, there was an increase in smoking quit intentions in both males (0.2 percentage points) and females (8.1 percentage points). However, there was a sharp decline in quit intentions among males (19.9 percentage points) and females (29.0 percentage points) during the second and third rounds of the GATS, as shown in Figure 4.

**Figure 5:**
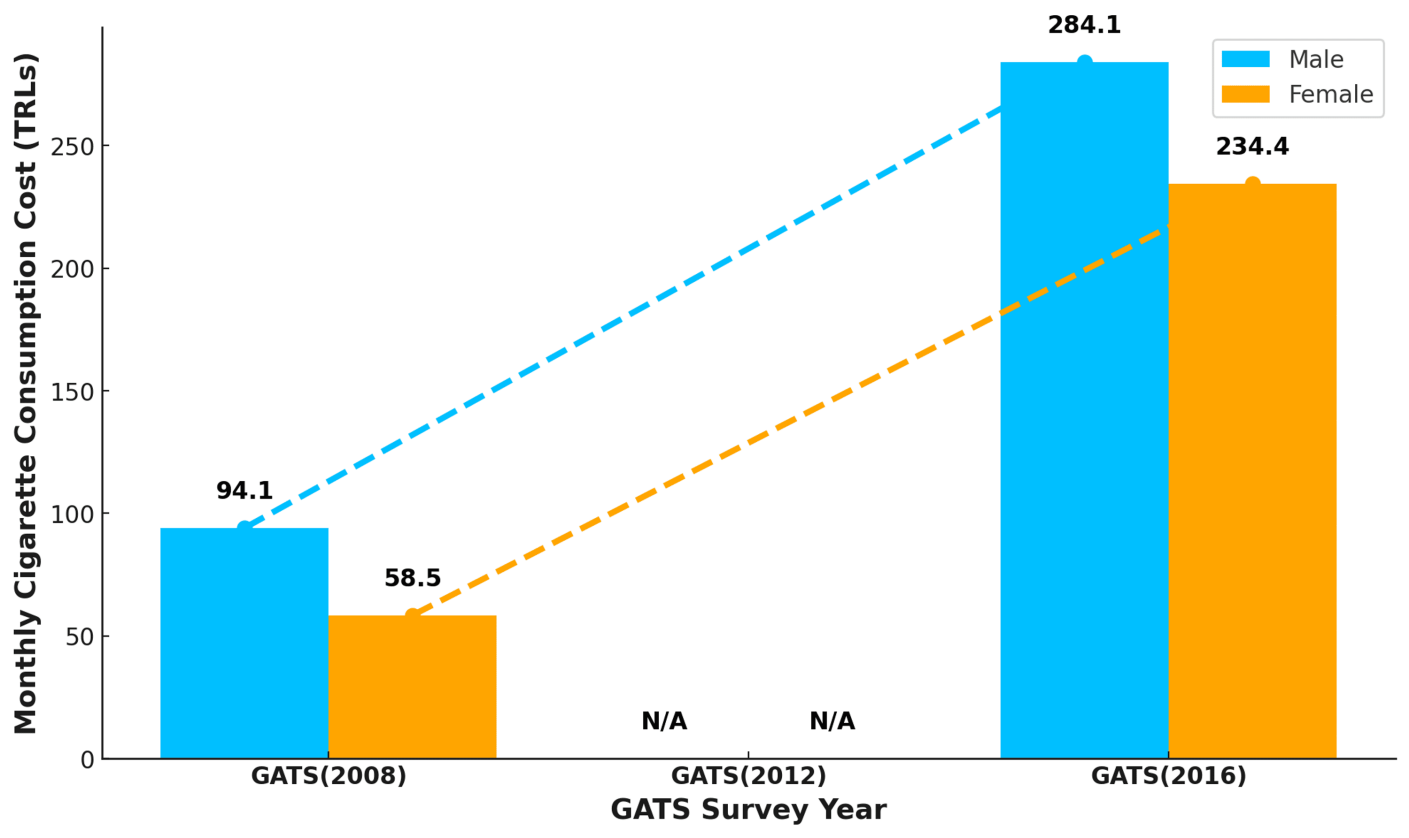
Trend in average monthly expenditures (in TRLs) on cigarettes among male and female cigarette smokers in Turkey N/A: Gender-stratified data for 2012 is unavailable. The numbers in the figure represent the TRL currency of Turkey. GATS: Global Adult Tobacco Survey, TRL: Turkish Lira

The average monthly expenditure on cigarettes among current smokers increased from 94.1 Turkish Lira (TRL) in 2008 to 284.1 TRL in 2016 among males (Figure 5). This upward trend was also observed for female current smokers. The average monthly expenses among males were higher than those among females during the first and third rounds of the GATS.

**Figure 6:**
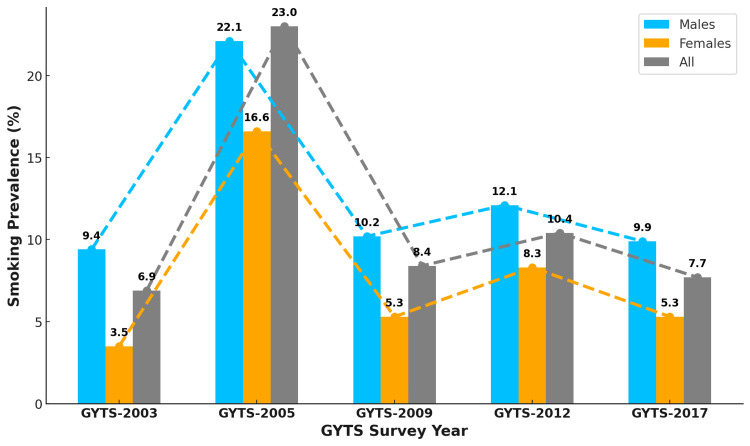
Trends in the prevalence of cigarette smoking among male and female youth in Turkey GYTS: Global Youth Tobacco Survey

The overall prevalence of cigarette smoking among youth increased significantly from the first round of the GYTS in 2003 (6.9%) to the second round in 2005 (23%). After this peak, there was a sharp decrease to 8.4% in 2009. The prevalence then slightly increased to 10.4% in 2012, followed by a decline to 7.7% in 2017, as depicted in Figure 6. Throughout all the GYTS rounds, males consistently had higher smoking rates than females. The large spike from 2003 to 2005, particularly among male youth, was followed by a more variable trend, peaking again in 2012 before declining by 2017.

Tobacco control legislations in Turkey

Law No. 4207 of 1996 on Preventing the Hazards of Tobacco Products and Restricting Advertisement and its amendment until 2008 aimed primarily at protecting public health from tobacco harm. It bans smoking in indoor public spaces, workplaces, schools, and trains [22]. Additionally, it prohibits all forms of tobacco advertising, promotion, and sponsorship. Sales of tobacco products to individuals under the legal minimum purchase age are criminal offenses. The law also mandates health warnings in public places and on tobacco packaging. Repeat offenses result in increasing fines. The law aims to reduce the negative impact of tobacco on society and improve public health. This comprehensive law was revised, and penalties were increased in 2011 and again in 2013, with the potential for business closures for repeat offenders, extending to food service establishments. The 2012 amendment expanded restrictions to include hookahs and related products.

In 2002, these efforts were complemented by Law No. 4733, which reorganized the tobacco market by setting up the Tobacco and Alcohol Market Regulatory Authority, which was tasked with overseeing the production, trade, and compliance of the tobacco and alcohol markets in Turkey [22]. These provisions also implemented stringent manufacturing standards for tobacco products and regulated imports and exports. The 2008 amendments introduced stricter penalties for the unauthorized production or sale of tobacco and alcohol, with fines of up to 500,000 Turkish Lira and potential imprisonment. Additionally, the amendments clarified the appointment and benefits of personnel within the Tobacco and Alcohol Market Regulatory Authority and established specific departments responsible for market regulation.

Turkey's Law No. 4822, enacted on March 6, 2003, amended the Consumer Protection Law to regulate commercial advertisements [11]. It mandates that advertisements be honest and truthful, and comply with legal and moral standards. Misleading advertisements, those that exploit vulnerable consumers or promote violence, are prohibited. The law also requires that advertisements be clearly identifiable as such.

Turkey became party to the WHO Framework Convention on Tobacco Control (FCTC) in 2005 and, in the same year, introduced the Regulation on the Production, Labeling, and Inspection of Tobacco Products to reduce the harmful effects of tobacco use by implementing strict rules on the content, labeling, and market monitoring of tobacco products. This regulation sets limits on the levels of tar, nicotine, and carbon monoxide in cigarettes and requires that health warnings cover 65% of the packaging. It also bans misleading information on product labels and prohibits the marketing of smokeless tobacco. Additionally, the regulation mandates that manufacturers submit annual reports detailing the ingredients used and their health impacts, ensuring transparency and consumer protection. Its amendment in 2008 further tightened emission limits and expanded labeling requirements. Later amendments in 2010 and 2012 increased the size of health warnings on packaging and imposed stricter market monitoring and compliance measures.

Within several years after the ban on loose cigarette sales came into effect in 2008, several other tobacco control laws were enacted in Turkey. Law No. 5326 [22] on Misdemeanors, in 2008, intended to fine those who smoke in any enclosed public space, public transportation, or other places noted as no smoking areas. Major amendments in 2008 of Law No. 4207 made it stricter in terms of regulating tobacco advertising and creating smoke-free environments. Circular No. 2009/13, which Prime Minister's Office (PMO) Circular 2009/13 introduced in 2009, extended the indoor smoking ban to all public and private spaces and called for public support with a view to ensuring strict compliance. Further regulations were brought out from 2012 to 2016, including the Tobacco and Alcohol Market Regulatory Authority (TAPDK) Decision No. 6896 and the Council Decision No. 7105 [11], the focus of which was toward the regulation of the ingredients in tobacco products, prohibiting specific harmful additives to set a yardstick as per public health standards. In this context, the National Tobacco Control Program (NTCP) Plan of Action 2015-2018 has been an overriding framework for reducing tobacco demand and supply, monitoring tobacco use, and evaluating the effectiveness of measures for tobacco control. In 2017, Decree No. 696 [11] introduced a key institutional change by dissolving TAPDK and assigning its enforcement duties to the Ministry of Agriculture and the Ministry of Health, which are now responsible for tobacco laws, health warnings, and product regulation (Table 2). Details of the Tobacco Control Law in Turkey are attached in the Appendices section (Supplementary 2) for reference.

**Table 2:** Timeline of the tobacco control laws and tobacco use prevalence by gender in Turkey from GATS and GYTS GATS: Global Adult Tobacco Surveys, GYTS: Global Youth Tobacco Survey, WHO FCTC: World Health Organization Framework Convention on Tobacco Control, TAPDK: Tobacco and Alcohol Market Regulatory Authority, PMO: Prime Minister's Office, NTCP: National Tobacco Control Program, HTPs: heated tobacco products Source: Global Tobacco Surveillance System Data (GTSSData) [21] and Turkey Tobacco Laws in Turkey [11,18]

Short name of legal provision or law	Year	GATS or GYTS survey conducted	Current cigarette smoking (male) (%)	Current cigarette smoking (female) (%)
Law No. 4207: Prevention and Control of the Harms of Tobacco Products	1996	-	-	-
Law No. 4733: The Market of Tobacco, Tobacco Products, and Alcohol	2002	-	-	-
Law No. 4822: Commercial Advertisements and Ads	2003	GYTS 1	9.40	3.50
Party to WHO FCTC	2005	GYTS 2	22.10	16.60
Regulation: Manufacture, type, labeling, and surveillance of tobacco products				
Law No. 5326: Misdemeanors	2008	GATS 1	45.80	14.90
Law No. 4207: Enforcement				
Law No. 4207: TAPDK				
Loose cigarette ban				
PMO Circular 2009/13 - Implementation of Law No. 4207	2009	GYTS 3	10.20	5.30
Production and trade of tobacco products	2010	-	-	-
MoH Guidance - Law No. 4207: Obligations of Provincial Authorities				
Sales and Presentations of Tobacco Products and Alcoholic Beverages	2011	-	-	-
TAPDK Decision No. 6896	2012	GATS 2	41.30	13
Council Decision No. 7105		GYTS 4	12.10	9.30
Tobacco products and alcoholic beverages law	2013	-	-	-
Regulation of business places with hookah smoking	2014	-	-	-
NTCP Plan of Action 2015	2015	-	-	-
Council Decision No. 9010				
Decision No. 2015-8353 Adjusting Tax Rates				
Penal Code of Turkey	2016	GATS 3	44.10	19.20
Decision No. 13016 on Cigarette Papers	2017	GYTS 5	9.90	5.30
Decree No. 696 - TAPDK dissolved and enforcement transferred to Agriculture and Health Ministry				
Formation of NTCP Committee	2018	-	-	-
Plain packaging	2019	-	-	-
Health warnings on hookah bottles	2020	-	-	-
Bans import of e-cigarettes and HTPs, and restriction on passengers				
Health warning covering 85% of the front and 100% of the back of the package of smoked tobacco products	2021	-	-	-

## Discussion

The trends in smoking behaviors in Turkey, before and after the implementation of the loose cigarette ban and other tobacco control policies, present a mix of promising and concerning patterns. The GATS and GYTS data show an initial decline in smoking rates following the introduction of these policies, indicating their immediate impact [23]. However, the subsequent increase in smoking prevalence suggests that maintaining these gains over the long term is challenging, a pattern consistent with findings from other countries [16].

Access to single cigarettes has been identified as a significant barrier to reducing smoking rates, particularly among vulnerable populations such as youth and low-income individuals [5]. The availability of loose cigarettes at affordable prices not only encourages tobacco use but also exacerbates health inequalities [5]. This issue is particularly concerning given the sharp rise in smoking rates among women, highlighting the need for gender-specific interventions. Similar trends in Western European countries further emphasize the necessity of targeted approaches to address smoking among women effectively [24].

Quit attempts and future quitting intentions, as indicated by GATS, have shown periods of decline, which contradicts the goals of tobacco control policies and warrants further investigation into the underlying causes. The significant increase in average monthly expenditures on cigarettes, as also reported in GATS, underscores the limitations of pricing and taxation policies as effective deterrents. This trend may reflect smokers' adaptation to price increases or a shift to more expensive tobacco products, a phenomenon observed in other high-income countries as well [25].

Although this study primarily focuses on the change of the loose cigarette sales ban on smoking behavior in Turkey, it is essential to consider the broader context. A comprehensive strategy should include rigorous policy enforcement, pricing and tax-based measures, and public health campaigns to raise awareness about the dangers of tobacco use [25].

Political instability and frequent leadership changes have slowed the progress of tobacco control policies in Turkey. Similar challenges are seen in countries such as Indonesia, where political transitions have been exploited by the tobacco industry to delay regulations [26], and in Brazil, where industry lobbying has impeded product display regulations and health warnings during periods of political uncertainty [27]. Despite these obstacles, Turkey has made progress, notably in enforcing smoke-free laws, banning tobacco advertising, and increasing taxes [11,12,23]. However, stronger enforcement and sustained political commitment remain crucial for ensuring the long-term success of these measures.

The relationship between political instability and the effective implementation of tobacco control policies in Turkey is intricate. Overcoming these challenges will require a sustained commitment from top leadership, robust legislation, effective enforcement mechanisms, and collaboration with civil society organizations and the public health community. These stakeholders are critical in advancing effective tobacco control measures, regardless of the political climate [23].

The marked increase in smoking rates among women points to significant gender disparities that demand gender-specific approaches. This concern is mirrored by studies in Western Europe, which highlight the need for targeted interventions to effectively reach women [24]. The rise can also be part of the usual tobacco epidemic curve where female smoking prevalence closely follows male smoking prevalence. Hassoy et al. (2021) confirm that "smoking among women in Turkey, especially among the lower socioeconomic classes, increased significantly between 2008 and 2016, reflecting the growing influences of economic inequities" [28]. These are driven by thematic marketing campaigns, taking the themes of freedom and glamour from tobacco companies [29]. These, in many high-income countries, are regulated; however, there is still targeting of the low-income region through social media and films, normalizing smoking. Biological factors and concerns about weight gain further complicate quitting, with misleading packaging such as slim cigarette designs perpetuating use [30]. These observations suggest the need for targeted, gender-specific public health interventions that address the social, cultural, and economic drivers of increasing smoking rates among women in these regions. In contrast, the decline in quitting attempts and future quitting intentions calls for a deeper examination of the factors driving these trends.

The high growth in average monthly cigarette expenditures also reflects the limitations of pricing and taxation policies as deterrents. This trend could indicate that smokers are either adapting to higher prices or switching to more expensive products, similar to patterns observed in high-income countries [25].

While this study concentrated on the impact of the loose cigarette ban on smoking patterns in Turkey, it is crucial to consider the broader context to fully understand tobacco control initiatives. These measures should include strict enforcement of existing tobacco control policies, the implementation of pricing and tax strategies to deter consumption, and comprehensive public health campaigns to educate the public about the hazards of tobacco use [25].

A study by Summers et al. (2022) highlighted the progress made in Turkey, particularly in the areas of smoke-free legislation, advertising bans, and increased taxation, but also emphasized the need for stronger enforcement and political commitment to sustain these gains [23]. Similarly, Calikoglu et al. explored the challenges posed by the tobacco industry's lobbying efforts and the variable political support that has allowed for a less urgent approach to implementing tobacco control measures in Turkey [12].

Fluctuations in youth smoking prevalence over time, as reported by the GYTS, reflect the interplay of sociocultural factors, policy measures, and the addictive nature of tobacco products. For instance, the increasing trends in smoking rates from 2003 to 2012 can be partially attributed to the strong marketing strategies of the tobacco industry, peer pressure, and the lack of comprehensive tobacco control measures during that period [24]. The significant increase in smoking rates among female youths from 3.5% in 2003 to 8.3% in 2012 is particularly concerning. The tobacco industry has been accused of deliberately targeting young women with gender-specific advertisements and packaging that exploit societal shifts portraying smoking as a symbol of emancipation and independence [24]. Data shows that the youth smoking prevalence decreased from 10.4% in 2012 to 7.7% in 2017, likely due to the implementation of comprehensive tobacco control measures during this period, including the ban on single-stick cigarettes, smoke-free public spaces, and advertising bans in line with WHO FCTC guidelines. However, the higher smoking rates among male youth (9.9% in 2017) suggest a need for gender-specific interventions. Factors such as socioeconomic status, smoking associated with perceived masculinity, and peer group influences should be considered in developing education campaigns and awareness efforts targeted at male youth [23]. The sharp rise in youth smoking between 2003 and 2005 could be attributed to aggressive tobacco marketing and increased cigarette accessibility during that time. Research shows that exposure to cigarette advertising can significantly influence adolescents' intentions to smoke, especially during formative years of self-concept development [2,30]. Further research is needed to fully understand the specific factors driving these trends in Turkey.

Strengthening the tobacco surveillance system by increasing the frequency of data collection and implementing biochemical validation to reduce reporting biases are key strategies for advancing tobacco control in Turkey. Public health campaigns should be tailored to Turkey's unique sociocultural and economic context, with a focus on addressing the growing smoking epidemic among women and young people. Raising taxes on all tobacco products, particularly loose cigarettes, will help discourage use, especially among vulnerable groups. Finally, to restrict youth access to loose cigarettes and curb their sales, strict enforcement of existing legislation through regular inspections is essential in the long term. These initiatives will require strong political will and collaboration with civil society.

The ecological nature of this study limits the ability to infer cause-and-effect relationships between the ban on loose cigarette sales and changes in smoking behavior. While this study provides valuable population-level insights, it cannot definitively attribute changes in smoking behavior to the ban on loose cigarettes. This limitation highlights the need for further statistical modeling or qualitative research to explore the causal mechanisms driving changes in smoking behavior following the ban.

Although this study did not utilize more advanced statistical models given the nature of the available data, future research could benefit from the application of interrupted time series analysis or multivariable regression models. Such models would allow researchers to more effectively extract the impact of the loose cigarette ban from other concurrent policy changes or external factors, including those related to market fluctuations or changes in cigarette accessibility. These would help isolate the specific impact of the ban by controlling for potential confounding factors and offering a more precise understanding of the temporal relationship between policy interventions and smoking behavior trends.

Self-reported data in this study may be subject to response biases, potentially affecting the validity of the findings. Without biochemical validation, such as cotinine levels, there is a possibility that the actual smoking prevalence may be underreported. Since cotinine validation was not available for the Turkish data, the absolute estimates should be interpreted with caution. However, the standardized data collection procedures across survey waves ensure that trends remain reliable for assessing changes over time. To address this limitation, future studies should incorporate objective measures or validation methods to enhance the accuracy of the results. Despite these challenges, the use of nationally representative data from GATS and GYTS strengthens the generalizability of this study. These trends offer valuable insights into the effectiveness of tobacco control measures and underscore the need for continuous reassessment and adaptation of policy in response to emerging challenges.

## Conclusions

Tobacco control measures in Turkey initially led to a drop in smoking rates and other positive trends. These improvements have not been sustained over time, reflecting the inherent complexities in tobacco control. This is alarming in view of the increasing rates of smoking among women and a corresponding decline in quit attempts and quit intentions. The history of a rapid increase in youth smoking also warrants close monitoring of youth tobacco use. This suggests that despite Turkey's tobacco control efforts making progress, there are still significant challenges that require comprehensive and full implementation of proven interventions in both letter and spirit.

Since the ban on loose cigarettes was introduced as part of a wider tobacco control law, alongside other legal and non-policy measures, it is difficult to determine its exact impact on smoking patterns in Turkey. That is why including clear impact assessment indicators in tobacco surveillance is so important. Tobacco control efforts should focus on gathering evidence to support the implementation of effective tobacco control laws and policies. Global tobacco surveillance systems need to track indicators that show the impact of different interventions, helping guide strategies not only in Turkey but also around the world. For Turkey to maintain progress in reducing tobacco use, it will need to stay committed to enforcing proven measures over the long term.

## Appendices

Supplementary 1: Python code

Python Code for Generating Cluster Bar Diagram With Trend Line

Smoking pattern from GATS (2008, 2012, 2016)

Import pandas as pd

# Load the Excel file

file_path = '/mnt/data/GATS Turkey 26 AUg.xlsx'

xls = pd.ExcelFile(file_path)

# Display the sheet names to understand the structure

sheet_names = xls.sheet_names

sheet_names

# Load the data from the first sheet

df = pd.read_excel(file_path, sheet_name='Sheet1')

# Display the first few rows to understand the structure of the data

df.head()

# Prepare the data by forward filling the NaN values in 'Smoking Pattern' column

df['Smoking Pattern'].fillna(method='ffill', inplace=True)

# Filter the data for males and females

df_male = df[df['Sex'] == 'Male'].dropna(axis=1, how='all').set_index('Smoking Pattern').drop(columns='Sex')

df_female = df[df['Sex'] == 'Female'].dropna(axis=1, how='all').set_index('Smoking Pattern').drop(columns='Sex')

# Convert the GATS data to numeric, since some values might be stored as strings

df_male = df_male.apply(pd.to_numeric, errors='coerce')

df_female = df_female.apply(pd.to_numeric, errors='coerce')

# Ensure all rows and columns are properly aligned

df_male.head(), df_female.head()

mport matplotlib.pyplot as plt

# Function to create cluster bar charts with bold trendlines for each smoking pattern

def plot_smoking_pattern_with_heavy_trendlines(smoking_pattern):

    data_male = df_male.loc[smoking_pattern]

    data_female = df_female.loc[smoking_pattern]

    plt.figure(figsize=(15, 9))  # Set the figure size to ensure higher resolution

    # Plotting bars with specified colors

    bars_male = plt.bar([x - 0.15 for x in range(len(data_male))], data_male, width=0.3, label='Male', alpha=0.7, color='deepskyblue')

    bars_female = plt.bar([x + 0.15 for x in range(len(data_female))], data_female, width=0.3, label='Female', alpha=0.7, color='orange')

    # Getting the positions and heights of the bars for trendlines

    positions_male = [bar.get_x() + bar.get_width() / 2 for bar in bars_male]

    top_male = [bar.get_height() for bar in bars_male]

    positions_female = [bar.get_x() + bar.get_width() / 2 for bar in bars_female]

    top_female = [bar.get_height() for bar in bars_female]

    # Plotting trendlines with even heavier line weight

    plt.plot(positions_male, top_male, linestyle='--', marker='o', color='deepskyblue', linewidth=5)

    plt.plot(positions_female, top_female, linestyle='--', marker='o', color='orange', linewidth=5)

    # Adding labels to the bars with font size equal to X and Y axis labels

    for i, bar in enumerate(bars_male):

        plt.text(bar.get_x() + bar.get_width() / 2, bar.get_height(), f'{bar.get_height():.1f}', ha='center', va='bottom', fontsize=20, fontweight='bold')

    for i, bar in enumerate(bars_female):

        plt.text(bar.get_x() + bar.get_width() / 2, bar.get_height(), f'{bar.get_height():.1f}', ha='center', va='bottom', fontsize=20, fontweight='bold')

    # Customizing the plot

    plt.xticks(range(len(data_male)), data_male.index, rotation=45, fontsize=20, fontweight='bold')  # Increased font size for X axis labels

    plt.yticks(fontsize=20, fontweight='bold')  # Increased font size for Y axis labels

    plt.xlabel('GATS Survey Year', fontsize=22, fontweight='bold')

    plt.ylabel(f'{smoking_pattern}', fontsize=22, fontweight='bold')

    plt.legend(loc='best', fontsize=20)

    plt.grid(axis='y')

    # Show plot

    plt.tight_layout()

    plt.show()

# List of smoking patterns to plot

smoking_patterns = df_male.index.tolist()

# Generate enhanced resolution plots for each smoking pattern with heavier trendlines

for pattern in smoking_patterns:

    plot_smoking_pattern_with_heavy_trendlines(pattern)

# Function to create cluster bar charts with bold trendlines and horizontal X-axis labels

def plot_smoking_pattern_with_horizontal_labels(smoking_pattern):

    data_male = df_male.loc[smoking_pattern]

    data_female = df_female.loc[smoking_pattern]

    plt.figure(figsize=(15, 9))  # Set the figure size to ensure higher resolution

    # Plotting bars with specified colors

    bars_male = plt.bar([x - 0.15 for x in range(len(data_male))], data_male, width=0.3, label='Male', alpha=0.7, color='deepskyblue')

    bars_female = plt.bar([x + 0.15 for x in range(len(data_female))], data_female, width=0.3, label='Female', alpha=0.7, color='orange')

    # Getting the positions and heights of the bars for trendlines

    positions_male = [bar.get_x() + bar.get_width() / 2 for bar in bars_male]

    top_male = [bar.get_height() for bar in bars_male]

    positions_female = [bar.get_x() + bar.get_width() / 2 for bar in bars_female]

    top_female = [bar.get_height() for bar in bars_female]

    # Plotting trendlines with even heavier line weight

    plt.plot(positions_male, top_male, linestyle='--', marker='o', color='deepskyblue', linewidth=5)

    plt.plot(positions_female, top_female, linestyle='--', marker='o', color='orange', linewidth=5)

    # Adding labels to the bars with font size equal to X and Y axis labels

    for i, bar in enumerate(bars_male):

        plt.text(bar.get_x() + bar.get_width() / 2, bar.get_height(), f'{bar.get_height():.1f}', ha='center', va='bottom', fontsize=20, fontweight='bold')

    for i, bar in enumerate(bars_female):

        plt.text(bar.get_x() + bar.get_width() / 2, bar.get_height(), f'{bar.get_height():.1f}', ha='center', va='bottom', fontsize=20, fontweight='bold')

    # Customizing the plot

    plt.xticks(range(len(data_male)), data_male.index, rotation=0, fontsize=20, fontweight='bold')  # X axis labels are horizontal now

    plt.yticks(fontsize=20, fontweight='bold')  # Increased font size for Y axis labels

    plt.xlabel('GATS Survey Year', fontsize=22, fontweight='bold')

    plt.ylabel(f'{smoking_pattern}', fontsize=22, fontweight='bold')

    plt.legend(loc='best', fontsize=20)

    plt.grid(axis='y')

    # Show plot

    plt.tight_layout()

    plt.show()

# Generate enhanced resolution plots for each smoking pattern with horizontal X-axis labels

for pattern in smoking_patterns:

    plot_smoking_pattern_with_horizontal_labels(pattern)

# Plotting the last diagram for "Monthly Cigarette Consumption Cost (Local Unit)" with trendline using only GATS(2008) and GATS(2016)

smoking_pattern = 'Montly Cigarette Consumption Cost (Local Unit)'

# Filter out the years GATS(2008) and GATS(2016) only

data_male = df_male.loc[smoking_pattern, ['GATS(2008)', 'GATS(2016)']]

data_female = df_female.loc[smoking_pattern, ['GATS(2008)', 'GATS(2016)']]

plt.figure(figsize=(15, 9))  # Set the figure size to ensure higher resolution

# Plotting bars with specified colors

bars_male = plt.bar([x - 0.15 for x in range(len(data_male))], data_male, width=0.3, label='Male', alpha=0.7, color='deepskyblue')

bars_female = plt.bar([x + 0.15 for x in range(len(data_female))], data_female, width=0.3, label='Female', alpha=0.7, color='orange')

# Getting the positions and heights of the bars for trendlines

positions_male = [bar.get_x() + bar.get_width() / 2 for bar in bars_male]

top_male = [bar.get_height() for bar in bars_male]

positions_female = [bar.get_x() + bar.get_width() / 2 for bar in bars_female]

top_female = [bar.get_height() for bar in bars_female]

# Plotting trendlines with even heavier line weight

plt.plot(positions_male, top_male, linestyle='--', marker='o', color='deepskyblue', linewidth=5)

plt.plot(positions_female, top_female, linestyle='--', marker='o', color='orange', linewidth=5)

# Adding labels to the bars with font size equal to X and Y axis labels

for i, bar in enumerate(bars_male):

    plt.text(bar.get_x() + bar.get_width() / 2, bar.get_height(), f'{bar.get_height():.1f}', ha='center', va='bottom', fontsize=20, fontweight='bold')

for i, bar in enumerate(bars_female):

    plt.text(bar.get_x() + bar.get_width() / 2, bar.get_height(), f'{bar.get_height():.1f}', ha='center', va='bottom', fontsize=20, fontweight='bold')

# Customizing the plot

plt.xticks(range(len(data_male)), data_male.index, rotation=0, fontsize=20, fontweight='bold')  # X axis labels are horizontal now

plt.yticks(fontsize=20, fontweight='bold')  # Increased font size for Y axis labels

plt.xlabel('GATS Survey Year', fontsize=22, fontweight='bold')

plt.ylabel(f'{smoking_pattern}', fontsize=22, fontweight='bold')

plt.legend(loc='best', fontsize=20)

plt.grid(axis='y')

# Show plot

plt.tight_layout()

plt.show()

Smoking Pattern from GATS (2003, 2005, 2009, 2012, & 2017)

# Load the new Excel file containing the youth data

youth_data_path = '/mnt/data/GYTS dataset.xlsx'

xls_youth = pd.ExcelFile(youth_data_path)

# Display the sheet names to understand the structure

sheet_names_youth = xls_youth.sheet_names

sheet_names_youth

# Load the data from the first sheet

df_youth = pd.read_excel(youth_data_path, sheet_name='Sheet1')

# Display the first few rows to understand the structure of the data

df_youth.head()

# Prepare the data by forward filling the NaN values in 'Smoking Pattern' column

df_youth['Smoking Pattern'].fillna(method='ffill', inplace=True)

# Filter the data for males, females, and all

df_youth_male = df_youth[df_youth['Sex'] == 'Males'].dropna(axis=1, how='all').set_index('Smoking Pattern').drop(columns='Sex')

df_youth_female = df_youth[df_youth['Sex'] == 'Females'].dropna(axis=1, how='all').set_index('Smoking Pattern').drop(columns='Sex')

df_youth_all = df_youth[df_youth['Sex'] == 'All'].dropna(axis=1, how='all').set_index('Smoking Pattern').drop(columns='Sex')

# Convert the GYTS data to numeric, since some values might be stored as strings

df_youth_male = df_youth_male.apply(pd.to_numeric, errors='coerce')

df_youth_female = df_youth_female.apply(pd.to_numeric, errors='coerce')

df_youth_all = df_youth_all.apply(pd.to_numeric, errors='coerce')

# Ensure all rows and columns are properly aligned

df_youth_male.head(), df_youth_female.head(), df_youth_all.head()

# Function to create cluster bar charts with bold trendlines, changing 'All' category to grey

def plot_youth_smoking_pattern_with_grey_trendlines(smoking_pattern):

    data_male = df_youth_male.loc[smoking_pattern]

    data_female = df_youth_female.loc[smoking_pattern]

    data_all = df_youth_all.loc[smoking_pattern]

    plt.figure(figsize=(15, 9))  # Set the figure size to ensure higher resolution

    # Plotting bars with specified colors

    bars_male = plt.bar([x - 0.2 for x in range(len(data_male))], data_male, width=0.2, label='Males', alpha=0.7, color='deepskyblue')

    bars_female = plt.bar([x for x in range(len(data_female))], data_female, width=0.2, label='Females', alpha=0.7, color='orange')

    bars_all = plt.bar([x + 0.2 for x in range(len(data_all))], data_all, width=0.2, label='All', alpha=0.7, color='grey')

    # Getting the positions and heights of the bars for trendlines

    positions_male = [bar.get_x() + bar.get_width() / 2 for bar in bars_male]

    top_male = [bar.get_height() for bar in bars_male]

    positions_female = [bar.get_x() + bar.get_width() / 2 for bar in bars_female]

    top_female = [bar.get_height() for bar in bars_female]

    positions_all = [bar.get_x() + bar.get_width() / 2 for bar in bars_all]

    top_all = [bar.get_height() for bar in bars_all]

    # Plotting trendlines with even heavier line weight

    plt.plot(positions_male, top_male, linestyle='--', marker='o', color='deepskyblue', linewidth=5)

    plt.plot(positions_female, top_female, linestyle='--', marker='o', color='orange', linewidth=5)

    plt.plot(positions_all, top_all, linestyle='--', marker='o', color='grey', linewidth=5)

# Adding labels to the bars with font size equal to X and Y axis labels

    for i, bar in enumerate(bars_male):

        plt.text(bar.get_x() + bar.get_width() / 2, bar.get_height(), f'{bar.get_height():.1f}', ha='center', va='bottom', fontsize=20, fontweight='bold')

    for i, bar in enumerate(bars_female):

        plt.text(bar.get_x() + bar.get_width() / 2, bar.get_height(), f'{bar.get_height():.1f}', ha='center', va='bottom', fontsize=20, fontweight='bold')

    for i, bar in enumerate(bars_all):

        plt.text(bar.get_x() + bar.get_width() / 2, bar.get_height(), f'{bar.get_height():.1f}', ha='center', va='bottom', fontsize=20, fontweight='bold')

    # Customizing the plot

    plt.xticks(range(len(data_male)), data_male.index, rotation=0, fontsize=20, fontweight='bold')  # X axis labels are horizontal now

    plt.yticks(fontsize=20, fontweight='bold')  # Increased font size for Y axis labels

    plt.xlabel('GYTS Survey Year', fontsize=22, fontweight='bold')

    plt.ylabel(f'{smoking_pattern}', fontsize=22, fontweight='bold')

    plt.legend(loc='best', fontsize=20)

    plt.grid(axis='y')

    # Show plot

    plt.tight_layout()

    plt.show()

# Generate enhanced resolution plots for each smoking pattern with heavier trendlines, 'All' category in grey

for pattern in smoking_patterns_youth:

    plot_youth_smoking_pattern_with_grey_trendlines(pattern)

Supplementary 2: Tobacco control laws in Turkey

Source: Tobacco Control Laws by Campaign for Tobacco-Free Kids (available at https://www.tobaccocontrollaws.org/legislation/turkey/laws)

Law No. 4207 of 1996: Prevention and Control of the Harms of Tobacco Products

Source URL: https://assets.tobaccocontrollaws.org/uploads/legislation/Turkey/Turkey-Law-No.-4207.pdf

Protection from tobacco hazards: This law focuses on the safety of the present and future generations from tobacco and its products. The risk from tobacco may be in a direct form as well as in a passive form.

Regulation of advertising: It outlaws the advertisement or any other form of tobacco exhibition, which would fire up its use or consumption.

Promotion of clean air: From a public health perspective, the law emphasizes that all individuals should have access to clean and breathable air, which is free from any kind of pollutants, especially tobacco smoke or dust.

Preventive measures and arrangements: In order to effectuate protection against the hazards of tobacco and advocate fresh air, adopting preventive measures and taking steps to attain them are necessary.

​​​​​​Law No. 4733 of 2002: The Market of Tobacco, Tobacco Products, and Alcohol

Source URL: https://assets.tobaccocontrollaws.org/uploads/legislation/Turkey/Turkey-Law-No.-4733.pdf

Reorganizing operations: The law focuses on restructuring the existing General Directorate that is accountable for tobacco, its products, salt, and alcohol operations.

Establishment of authoritative bodies: The Tobacco and Alcohol Market Regulatory Authority was established to regulate the market and check its compliance with the ordinance.

Board duties and authorities: The law traces the activities and responsibilities of the Tobacco and Alcohol Market Regulatory Board.

Regulation of market activities: A thorough guideline was established for the regulation of the manufacture and marketing of tobacco and its products within Turkey.

Auction price determination

Law No. 4822 of 2003: Commercial Advertisements and Ads

Source URL: https://assets.tobaccocontrollaws.org/uploads/legislation/Turkey/Turkey-Law-No.-4822.pdf

Integrity in advertising and consumer protection: The advertisements should not be misleading and should also abide by regulations forwarded by the concerned authorities. The advertisements should not be a source of asymmetrical information.

Safety and health concerns: Advertisements that jeopardize life and property or that pose a threat to public health are prohibited.

Prevention of violence and crime: Advertisements that promote violence and criminal activities are prohibited to maintain public order.

Protection of vulnerable groups: It should restrict the exploitation of the elderly, minors, and specially-abled individuals and also to clinch on the principle of ethical content toward the susceptible population.

2005 Regulation: Manufacture, Type, Labeling, and Surveillance of Tobacco Products

Source URL: https://assets.tobaccocontrollaws.org/uploads/legislation/Turkey/Turkey-Regs-on-Manufacture-Labeling-and-Inspection.pdf

Comprehensive definitions: It lays emphasis on the process and varieties of tobacco and its products for regulatory purposes.

Health and safety regulations: It lays stress on the public about the societal and medical consequences of the consumption of tobacco and its by-products.

Mandatory health warnings

Law No. 5326 of 2008: Misdemeanors

Source URL: https://assets.tobaccocontrollaws.org/uploads/legislation/Turkey/Turkey-Law-No.-5326.pdf

Article 39: (1) A handsome amount of fine shall be levied upon people who are found smoking in enclosed public spaces. (2) A fine of 50 Turkish Lira shall be imposed on those who are caught in the act of consumption of tobacco on public transport. (3) People who would violate the "no smoking" zones would also be fined.

Law No. 4207 of 2008: Enforcement

Source URL: https://assets.tobaccocontrollaws.org/uploads/legislation/Turkey/Turkey-Law-No.-4207.pdf

Protection from hazards: To follow the necessary measures and make specific arrangements to safeguard all individuals and also to reduce the harmful effects of tobacco.

Regulation of advertising and promotion: To engage in a responsible way when advertising and promoting the products.

Ensuring clean air

Law No. 4207 of 2008: TAPDK

Source URL: https://assets.tobaccocontrollaws.org/uploads/legislation/Turkey/Turkey-Law-No.-4207.pdf

In smoke-free places, it should be made mandatory to display form and content of legal warnings and rules related to the placement of legal warnings.

In tobacco retail points of sale, rules related to legal warnings should be displayed.

In areas designated for tobacco use, rules related to health warnings are required to be displayed.

PMO Circular 2009/13: Implementation of Law No. 4207

Source URL: https://assets.tobaccocontrollaws.org/uploads/legislation/Turkey/Turkey-Circular-200913.pdf

Comprehensive indoor ban: In order to protect the health of the public, a ban should be imposed on the use of tobacco in all indoor as well as both public and private spaces.

Broad implementation scope: It holds true in all settings, such as educational, healthcare, and community.

Public support and compliance: It stimulates the support of the masses and also acknowledges their support for the aim toward a healthier society by following tobacco control measures.

Enforcement and accountability: It abides by the enforcement of the law and ensures strict enforcement of the law with administrative companions and accountability for subordinates.

2010 MoH Guidance (Law No. 4207): Obligations of Provincial Authorities

Source URL: https://assets.tobaccocontrollaws.org/uploads/legislation/Turkey/Turkey-MoH-Guidance-on-Inspections-Enforcement.pdf

Enhanced inspection protocols: It should focus on both indoor and outdoor areas to ensure detailed compliance.

Training and inclusion of police: It should focus on the training of inspectors, including the representatives comprising of police in their teams for efficient and effective enforcement.

Strict enforcement and accountability: Emphasis should be placed on prioritizing sudden inspections to ensure genuine compliance. Imposing strict penalties for violators should be required, as well as holding public officials accountable for their duties.

Coordination with authorities

2012 TAPDK Decision No. 6896

Source URL: https://assets.tobaccocontrollaws.org/uploads/legislation/Turkey/Turkey-TAPDK-Decision-No.-6896.pdf

• Assessment of ingredient notification and toxicological data found on the data tables

• Products for export

• Market supply of the products

• Organization of the Scientific Commission, their principles, and working methods

2012 Council Decision No. 7105

Source URL: https://assets.tobaccocontrollaws.org/uploads/legislation/Turkey/Turkey-Decision-No.-7105-on-Ingredients-Disc.pdf

Prohibition of specific additives: It would make sure that all the harmful contents are excluded from the finished products.

Scope of application: The provision would apply to all tobacco products produced within Turkey, imported into Turkey, and supplied to the Turkish market.

Regulatory compliance: It regulates the compliance of all tobacco product manufacturers and importers to comply with the prohibition of specific additives, which in turn aims to increase the safety and health standards of tobacco products.

The NTCP Plan of Action 2015

Source URL: https://assets.tobaccocontrollaws.org/uploads/legislation/Turkey/Turkey-Plan-of-Action-2015-2018.pdf

• The steps that are to be undertaken toward the scaling down of tobacco demand

• The steps that are to be taken toward decreasing the supply of tobacco

• The monitoring, evaluation, and implementation of tobacco and tobacco products and national tobacco control program action plan

2015 Council Decision No. 9010

Source URL: https://assets.tobaccocontrollaws.org/uploads/legislation/Turkey/Turkey-Decision-No.-9010-re-Menthol.pdf

Comprehensive ban on menthol and derivatives: An extensive ban of menthol and its derivatives in both cut tobacco and hand-rolled products should be imposed.

Broad application: This is applicable to both in-house-grown produce and imported tobacco products.

Regulated areas: The ban covers add-ons in raw materials, cigarette paper, adhering spots, ink, tip paper, aluminum foil, and packaging.

2015 Decision No. 2015-8353 Adjusting Tax Rates

Source URL: https://assets.tobaccocontrollaws.org/uploads/legislation/Turkey/Turkey-Decision-No.-2015-8353-on-Taxes-native.pdf

Purpose and scope: The Decree aims to determine the rates of value-added tax (VAT), special consumption tax (SCT), and tobacco fund amounts for certain goods.

Legal basis: The Decree is based on Article 28 of the Value Added Tax Law (Law No. 3065) dated 25/10/1984, Article 12 and Provisional Article 6 of the Special Consumption Tax Law (Law No. 4760) dated 6/6/2002, and Provisional Article 1 of the Law on the Liquidation of Certain Funds (Law No. 4629) dated 21/2/2001.

Amendments to VAT list: New items added to the (I) list of the Decision on Determining the Rates of Value Added Tax to be Applied to Goods and Services (Decision No. 2007/13033 dated 24/12/2007).

Repeal of item 23 in the section "(B) OTHER GOODS AND SERVICES" of the (II) list annexed to the same Decision.

New items added to VAT list: Animal feeds including full-fat soy, bran, rapeseed, fish meal, meat meal, bone meal, blood meal, tapioca (cassava), sorghum, and all kinds of compound feeds (excluding cat and dog foods); other animal feeds such as straw, feed turnip, fodder beet, root feeds, dry hay, alfalfa, vetch, sainfoin, silage corn, clover, fodder cabbage, feed peas, and similar animal feeds (including processed green and dry roughage in pellet form or with a binder used as per seasonal needs); and fertilizers registered by the Ministry of Food, Agriculture, and Livestock and the delivery of raw materials contained in these products to fertilizer producers.

Determination of SCT rates and amounts: It specifies the tax rates, minimum specific, and specific tax amounts for goods listed in the (A) and (B) schedules of the (III) list annexed to the Special Consumption Tax Law.

2017 Decision No. 13016 on Cigarette Papers

Source URL: https://assets.tobaccocontrollaws.org/uploads/legislation/Turkey/Turkey-Decision-No.-13016.pdf

• Determining the readiness with technical regulations

• The ingredients of the product

• The style of packaging for market supply

• The physical features of the product

• The products that are intended for export

• The law enforcement

2017 Decree No. 696: TAPDK Dissolved and Enforcement Transferred to Agriculture and Health Ministry

Source URL: https://assets.tobaccocontrollaws.org/uploads/legislation/Turkey/Turkey-Decree-No.-696-native.pdf

Scope of application: The Decree applies to relatives of individuals who lost their lives within the scope of the Anti-Terror Law numbered 3713 dated 12/4/1991.

It specifically includes conscripts performing their military service and civilians covered by subparagraph (j) of the first paragraph of Article 21 of Law No. 3713.

Eligible relatives: Conscripts and civilians: all children and full siblings (from the same mother and father) of conscripts performing their military service and civilians covered by the Anti-Terror Law; and public officials: one child or full sibling (from the same mother and father) of public officials, including security guards.

Exemption and discharge provisions: Eligible relatives are not to be conscripted unless they wish to be. Those who are already conscripted shall be discharged upon their request. The exemption or discharge is granted without mandatory conditions, based purely on the wish of the eligible relatives.

Determination of exemption: The procedure to determine which sibling will be exempted from military service is regulated in the first paragraph of the relevant subparagraph.

This process ensures clarity and fairness in the exemption determination among eligible siblings.

Service location for non-exempt relatives: For those who are not covered by the exemption or who do not wish to benefit from the exemption, the places where they will perform their military service will be specified in the relevant directive.

This ensures that the military service of non-exempt or non-benefiting relatives is properly managed and organized.
